# [Expression of Concern] Long non-coding RNA SNHG20 promotes bladder cancer via activating the Wnt/β-catenin signalling pathway

**DOI:** 10.3892/ijmm.2026.5737

**Published:** 2026-01-21

**Authors:** Qingsong Zhao, Saiyue Gao, Qingyan Du, Ye Liu

Int J Mol Med 42: 2839-2848, 2018; DOI: 10.3892/ijmm.2018.3819

Following the publication of this paper, it was drawn to the Editor's attention by a concerned reader that the control GAPDH western blots featured in Fig. 5A and B on p. 2845 were apparently the same (even though it is possible that the experiments portrayed in these figure parts were performed under the same experimental conditions).

The authors have been contacted by the Editorial Office to offer an explanation for the matter described above, although up to this time no response from them has been forthcoming. Owing to the fact that the Editorial Office has been made aware of potential issues surrounding the scientific integrity of this paper, we are issuing an Expression of Concern to notify readers of this potential problem while the Editorial Office continues to investigate this matter further.

